# Factors Associated with Clinically Important Changes in Quality of Life of Heart Failure Patients: The QUALIFIER Prospective Cohort Study

**DOI:** 10.3390/jcm14145079

**Published:** 2025-07-17

**Authors:** Irene Marques, Milton Severo, António Gomes Pinto, Cândida Fonseca, Henrique Cyrne Carvalho

**Affiliations:** 1 Serviço de Medicina Interna, Centro Hospitalar Universitário de Santo António (CHUdSA), Unidade Local de Saúde de Santo António, 4099-001 Porto, Portugal; 2 Unidade Multidisciplinar de Investigação Biomédica, Instituto de Ciências Biomédicas de Abel Salazar (ICBAS), 4050-313 Porto, Portugal; aagrpinto@gmail.com (A.G.P.); hjcarvalho@icbas.up.pt (H.C.C.); 3 ITR-Laboratory for Integrative and Translational Research in Population Health, 4050-600 Porto, Portugal; msevero@icbas.up.pt; 4 Centro Académico Clínico ICBAS-Santo António (CAC ICBAS-Santo António), 4050-313 Porto, Portugal; 5 EPIUnit, Instituto de Saúde Pública da Universidade do Porto, 4050-600 Porto, Portugal; 6 Clínica de Insuficiência Cardíaca, Serviço de Medicina Interna, Unidade Local de Saúde de Lisboa Ocidental, 1449-005 Lisboa, Portugal; mcandidafonseca@gmail.com; 7 NOVA Medical School, Faculdade de Ciências Médicas, Universidade NOVA de Lisboa, 1169-056 Lisboa, Portugal

**Keywords:** quality of life, heart failure, real-world

## Abstract

**Background/Objectives**: We aimed to identify the factors associated with clinically important changes in quality of life (QoL) of real-world heart failure (HF) patients. **Methods**: This is a single-centre, prospective cohort study including 419 patients at an HF clinic between January 2013 and February 2020. QoL was assessed regularly using Minnesota Living with Heart Failure Questionnaire (MLHFQ). We used five nested linear mixed-effects models to account for QoL measurements between patients and within-patient. Models were adjusted for time, sociodemographic factors, comorbidities, self-care adherence, and HF severity factors. **Results**: Median age was 78 years, 54.4% of patients were female, and 49.6% had left ventricle ejection fraction ≥ 50%. At baseline, 62.5% of patients were New York Heart Association (NYHA) class II. Median N-terminal-pro-B type natriuretic peptide level was 1454 pg/mL. Mean MLHFQ total score at baseline was 25 points (95%CI: 22.97–27.60). Having an implanted cardiac resynchronization therapy-pacemaker (CRT-P) was associated with moderate to large improvement in QoL (−13.55 points, 95%CI: −22.45–−4.65). NYHA class II and estimated glomerular filtration rate < 30 mL/min/1.73 m^2^ were associated with small to moderate QoL deterioration (9.74 points, 95%CI: 6.74–12.75 and 5.82 points, 95%CI: 1.17–10.47, respectively). NYHA classes III or IV and a recent HF hospitalization were associated with large to very large QoL deterioration (28.39 points, 95%CI: 23.82–32.96; 60.59 points, 95%CI: 34.46–86.72; and 26.91 points, 95%CI: 21.80–32.03, respectively). **Conclusions**: CRT-P implantation, NYHA class and HF hospitalization are associated with the most clinically important QoL changes.

## 1. Introduction

Heart failure (HF) is a multifaceted and life-threatening syndrome characterized by significant morbidity and mortality and poor functional capacity [1]. HF affects multiple aspects of a patient’s life (e.g., physical, mental/emotional, and social), resulting in a reduction in quality of life (QoL) [2]. For patients with such chronic and progressive disease, QoL is just as important as prolonging survival, especially considering that HF primarily affects older adults. Worldwide, Portugal is one of the countries with the highest proportions of elderly people, reported to be 24.11% in 2023, according to the World Bank [3]. In Portugal, one in three individuals aged 70 years or older has HF [4]. Therefore, it is urgent to better understand patients’ perspectives to move toward patient-centred HF management [2].

QoL has been used as a relevant patient-reported outcome and independent endpoint in HF trials [5,6,7]. Additionally, QoL assessment using a validated questionnaire was defined as a quality indicator for the management of HF patients [8]. The magnitude of within-patient changes in QoL scores that are clinically meaningful to HF patients has been studied [9]. Clinically important changes in QoL have been proposed, to identify patients who experience improvement or deterioration. In March 2025, the European Society of Cardiology reported a position statement on QoL in HF that aims to foster the systematic integration of the patient’s standpoint in HF care [10].

The QUALIFIER (QUALIty oF lIfe in hEart failuRe) study aims to assess QoL using a validated HF-specific questionnaire in a real-world cohort of patients admitted to an HF multidisciplinary clinic, and to identify the factors associated with clinically important changes in QoL.

## 2. Materials and Methods

### 2.1. Study Design and Clinical Setting

This single-centre, prospective, open cohort study included all patients admitted to a multidisciplinary HF outpatient clinic between January 2013 and February 2020, with at least one QoL assessment by the Minnesota Living with Heart Failure Questionnaire (MLHFQ). HF diagnosis and classification followed the universal definition and classification of HF [11]. The HF clinic inclusion and exclusion criteria, and management program, have been described previously [12,13]. Briefly, at every visit, the patient was observed by a nurse and an internist. The nurse conducted motivational interviews, assessing and promoting HF literacy and self-care behaviour through a prespecified checklist. New York Heart Association (NYHA) functional class was assessed by the physician. The Six-Minute Walking Test was performed, and the Hospital Anxiety and Depression Scale was administered regularly to assess functional capacity, as well as anxiety and depression. Polysomnography was requested for most patients. Patients were invited to participate in a cardiac rehabilitation program.

A holistic approach was used, concerning the diagnosis, management and treatment of patient comorbidities, targeted alongside HF care. Nurse and physician evaluations were documented in the electronic medical records immediately after patient consultation using prespecified structured records. The MLHFQ total score was made available to the professionals immediately. The HF multidisciplinary team meet weekly to decide investigation and/or treatment plans, namely cardiac device implantation.

The study adhered to the recommendations of the Declaration of Helsinki. The study protocol was approved by the local ethics committee and institutional review boards, with reference number 2017.040 (040-DEFI/040-CES).

### 2.2. QoL Evaluation and Data Collection

QoL was assessed at 3 months, 9 months, and 2 years after admission, and then annually. Patients who were unable to understand written questionnaires and those with dementia were excluded. Patients with dementia were identified using clinical records where the dementia diagnosis was previously established and through cognitive assessment using the Mini-Cog and Mini Mental State Examination. The Mini-Cog test was part of the HF management program and was applied to all patients during their first visit to the HF clinic and, subsequently, every year. Patients with an abnormal Mini-Cog test were further evaluated using the Mini Mental State Examination to identify dementia. The inability to understand written questionnaires was assessed by the nurse administering the questionnaires.

The MLHFQ is a validated instrument for evaluating HF-specific QoL; it consists of 21 questions specific to HF and appraises QoL over the previous four weeks. Questions are answered using a Likert scale ranging from 0–5; 0 indicates that the question has no impact on the patient or is not applicable, whereas 5 indicates the greatest adverse impact. The score range is 0–105 points; a higher score represents a poorer QoL. The MLHFQ was validated in the Portuguese language [14]. The translation and validation of the Portuguese version of MLHFQ used in this research were finalized before 2013, even though its validation in a larger sample of HF patients was published more recently.

The baseline was defined as the first MLHFQ administration. Demographic data were collected at HF clinic admission. Comorbidities were collected at clinical admission or were diagnosed at follow-up. Other variables were collected: HF aetiology; NYHA class; systolic blood pressure (SBP); heart rate; heart rhythm on the last electrocardiogram; last transthoracic echocardiogram parameters: left ventricle ejection fraction (LVEF), right ventricle (RV) systolic dysfunction, pulmonary artery systolic pressure (PASP), and severe tricuspid regurgitation; HF literacy; self-care behaviour; poor adherence to recommendations and treatments; HF hospitalizations (HFHs); urgent HF visits; last laboratory results: haemoglobin, blood creatinine, estimated glomerular filtration rate (eGFR) according to the Chronic Kidney Disease Epidemiology Collaboration (CKD-EPI) creatinine equation and N-terminal-pro-B type natriuretic peptide (NT-proBNP); HF medications and devices; completed cardiac rehabilitation program; and nocturnal non-invasive ventilation.

### 2.3. Study Outcomes

The primary outcome was to measure the impact of demographic factors, comorbidities, and other clinical variables on the total score of the MLHFQ. The effect of each factor on QoL was measured between patients and within-patient, by measuring QoL changes over time.

The secondary outcome was to identify factors that resulted in clinically important changes in QoL. Clinically important changes were classified using previously proposed ranges of 5, 10 or 15 points to refer to small-to-moderate, moderate-to-large, and large-to-very-large changes in QoL, respectively [9,10].

### 2.4. Statistical Analysis

The categorical variables are presented as frequencies and percentages. The normality of the data was assessed through visual graphic verification and the Shapiro–Wilk test. Non-skewed continuous variables were described using means and standard deviations (SDs). Variables with skewed distributions were described using medians and interquartile ranges (IQRs). Considering the longitudinal nature of the data, we used linear mixed effect (LME) models with random intercept and random time slope to account for the dependence of score measurements within-patient. Five LME models were parameterized: Model 1 was adjusted for time and time squared; Model 2 added adjustments for age, sex and self-reported low income; Model 3 included adjustments for chronic kidney disease (CKD), anaemia at HF admission, chronic pulmonary condition, anxiety, and depression; Model 4 added adjustments for self-care adherence; and Model 5 incorporated adjustments for NYHA class and NT-proBNP. The nested models were constructed considering structure: i.e., Model 2 included sociodemographic factors, Model 3 added comorbidities, Model 4 included self-care adherence and Model 5 incorporated HF severity. Each model included a small number of variables to reduce the probability of multicollinearity. We assumed that missing data occurred at random (Missing at Random—MAR). As mixed-effects models provide valid estimates under the MAR assumption and have demonstrated robustness in the presence of incomplete data, individuals with fewer time points were retained in the analysis.

To identify clinically important changes in QoL, the magnitude of the impact of each factor on the MLHFQ score was obtained by comparing the fixed regression coefficient with 5, 10 and 15 when categorical variables were considered. For continuous variables, the fixed regression coefficient was multiplied by the SD of each variable, and the results were compared with 5, 10 and 15.

A significance level of 5% was assumed. Statistical analysis was performed using R version 4.3.0 software (R Foundation for Statistical Computing, Vienna, Austria).

## 3. Results

### 3.1. Patient Demographic and Clinical Characteristics

A total of 595 patients were observed at the HF clinic during the study period. Follow-up was less than 3 months for 79 patients and 97 patients met the exclusion criteria for MLHFQ administration. The remaining 419 patients composed the study sample. At baseline, patients had a median follow-up of 3 months. During the study, 70 (16.7%) patients died. The average follow-up was 22 months, and the maximum was 84 months. At follow-up, there were 348 MLHFQ observations between 2 and 5 months, 244 between 8 and 12 months, 115 between 22 and 27 months, and 167 observations between 27 and 84 months of follow-up.

The patients’ characteristics are presented in Table 1 and in more detail in Supplementary Table S1.

Briefly, the patients’ median age was 78 years, with a female predominance (54.4%). At HF clinic admission, 49.6% of the patients had preserved LVEF (≥50%), and 40.6% had a reduced LVEF (≤40%). The HF aetiology was primarily hypertensive (42.5%), ischemic (38.9%), and/or valvular (34.8%). At baseline, most patients had NYHA class II symptoms (62.5%), and the median NT-proBNP level was 1454 pg/mL. Most patients had several cardiovascular and non-cardiovascular comorbidities. Hypertension, dyslipidaemia, iron deficiency, a history of atrial flutter or fibrillation, CKD, diabetes mellitus and anaemia were the most frequent comorbidities, each of which was present in more than half of the patients. At baseline, 60.6% of the patients were taking at least one renin–angiotensin–aldosterone system inhibitor (RAASi). Only 33.9% of patients adhered to HF self-care behaviour, 17.2% had a cardiac device implanted, and 9.3% had had HFH in the previous 28 days (Table 1). In the follow-up, a total of 16 patients received a CRT-P device.

### 3.2. QoL Trajectory

**Figure 1:**
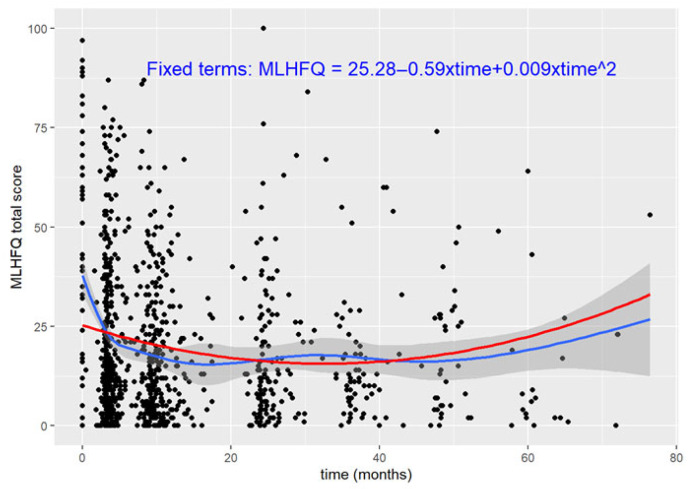
MLHFQ total score trajectory over time. The blue line represents the generalized additive model with smooth term for time. The red line indicates the linear and fixed terms for time of the LME model.

The mean MLHFQ total score at baseline was 25 points (95%CI: 22.97–27.60). We found a nonlinear association between scores and time. The generalized additive model revealed higher scores at baseline and a rapid decline, stabilizing at 10 months of follow-up. The time trend of the score was a decrease of 0.59 points/month. The inclusion of a quadratic term (β = 0.009) to account for that nonlinearity resulted in an inverse J-curve. Both the linear and quadratic terms were statistically significant (95%CI: −0.80–−0.38 and 95%CI: 0.005–0.013, respectively) (Figure 1).

### 3.3. Factors Associated with Significant QoL Changes

The results of the univariate analysis (Model 1) and multivariate analysis (Models 2 to 5) are presented in Table 2 and Table 3. The detailed results of the statistical analysis are reported in the Supplementary Tables S2–S4.

Multivariate analysis adjusted for patient characteristics is represented in Model 2 and the adjustment for comorbidities is represented in Model 3. Model 4 was adjusted for self-care adherence and Model 5 was adjusted for two surrogate markers of HF severity, NYHA class and NT-proBNP level (Table 2 and Table 3).

**Figure 2:**
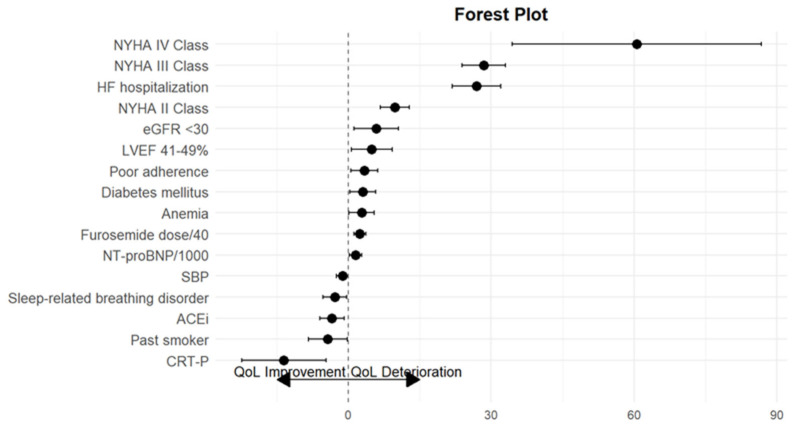
MLHFQ total score changes and factors associated with significant QoL improvement (on the left) and deterioration (on the right).

The final model (Model 5) revealed that past smokers, patients with sleep-related breathing disorder, those with higher SBP, those taking an ACEi, and those with an implanted CRT-P had significantly better QoL (Figure 2).

The only factor associated with clinically important improvement in QoL was having an implanted CRT-P (−13.55 points, 95%CI: −22.45–−4.65). Considering that only 16 patients had an implanted CRT-P, we performed a power simulation based on 200 simulations, using the simr package in R. We found that the estimated power to detect the effect of CRT-P was 44% (95% CI: 37.0-51.2%). Although this is below the conventional 80% threshold, it still provides some statistical power to detect an effect of CRT-P, suggesting that it is not a random effect.

Patients with diabetes, anaemia, NYHA class II to IV, LVEF between 41% and 49%, poor adherence, HFH in the previous 28 days, eGFR < 30 mL/min/1.73 m^2^, high NT-proBNP and high furosemide dose were associated with significantly worse QoL (Figure 2). The factors associated with clinically important deterioration in QoL were NYHA class (9.74 points, 95%CI: 6.74–12.75; 28.39 points, 95%CI: 23.82–32.96; and 60.59 points, 95%CI: 34.46–86.72, for classes II, III and IV, respectively), HFH (26.91 points, 95%CI: 21.80–32.03) and eGFR < 30 mL/min/1.73 m^2^ (5.82 points, 95%CI: 1.17–10.47) (Table 2 and Table 3, Model 5). The detailed results of the statistical analysis are reported in Supplementary Tables S2–S4. We performed sensitivity analyses using several factors, such as HF literacy, self-care literacy, self-care adherence, exercise adherence, all-cause hospitalizations and HFH, which did not change the results.

**Figure 3:**
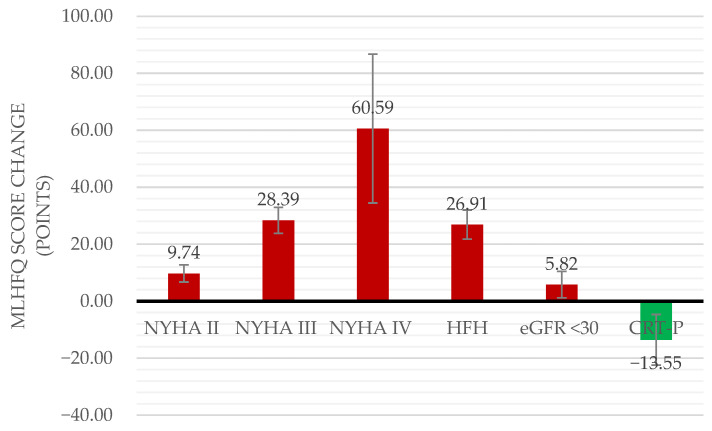
Factors associated with clinically important deterioration (red bars) or improvement (green bar) in MLHFQ total score.

CRT-P implantation was associated with a moderate-to-large improvement in QoL. NYHA class II and eGFR < 30 mL/min/1.73 m^2^ were associated with small-to-moderate QoL deterioration, whereas NYHA class III or IV and a recent HFH were associated with large-to-very-large QoL deterioration (Figure 3).

**Figure 4:**
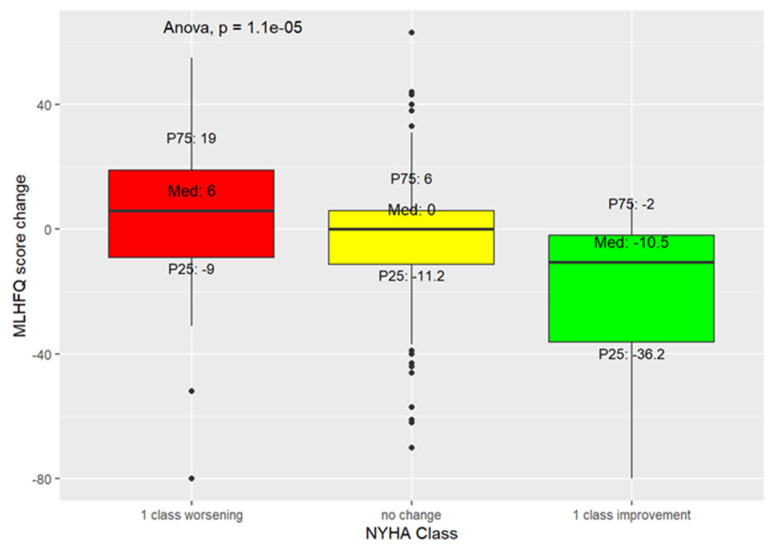
MLHFQ median score changes resulting from one NYHA class deterioration (red box), no change (yellow box), or one NYHA class improvement (green box).

Furthermore, we found that in patients with NYHA classes I-III, the improvement or worsening of one NYHA class was associated with a median decrease of 10.5 points (P25 =−36.2, P7 = −2) and an increase of 6 points (P25 =−9, P7 = 19) in MLHFQ score, respectively (Figure 4).

## 4. Discussion

This study reports QoL assessment of a cohort of 419 HF outpatients admitted to a multidisciplinary HF clinic over more than seven years. QoL assessment was part of the structured follow-up program and routine clinical practice. After analysing a comprehensive group of demographic and clinical factors, the only factor associated with clinically important improvement in QoL was the implantation of a CRT-P, which resulted in a moderate-to-large improvement. NYHA class II and an eGFR < 30 mL/min/1.73 m^2^ were associated with small-to-moderate clinically important QoL deterioration, whereas NYHA class III or IV and a recent HFH were associated with large-to-very-large clinically important QoL deterioration.

Most patients in the study were female, had LVEF ≥ 50%, and had hypertensive and/or ischaemic heart disease, reflecting the Portuguese HF population. In Portugal, HF prevalence is twice as high in women as it is in men, and 93% of HF patients aged 50 years or older have LVEF ≥ 50% [4]. Not surprisingly, we found a high burden of comorbidities in an elderly population with preserved LVEF [15]. At baseline, most patients were NYHA class II, had high levels of NT-proBNP, and were taking a RAASi and/or a beta-blocker. Notably, this study began before the approval of sacubitril/valsartan and sodium–glucose co-transporter 2 inhibitors (SGLT2i) for treatment of HF.

The mean MLHFQ total score at baseline was 25 points, and the score improved over time, until 10 months of follow-up. This finding is better than that in other reports [16,17], and is expected in patients under optimized care and follow-up [18,19].

We found that some factors traditionally associated with better or worse QoL in HF patients lost their impact on QoL when other factors were taken into consideration in the LME models [20,21]. The positive effect of male sex on QoL was captured after adjustment for NYHA and NT-proBNP. This probably reflects fewer symptoms reported by men, improving NYHA. We observed a similar pattern with self-care adherence and cardiac rehabilitation. The improvement in symptoms is the likely explanation for the improved QoL of patients who adhere to self-care and/or exercise. In contrast, the negative effect of depression on QoL was captured after adjustment for NYHA and NT-proBNP. Probably, worse symptoms in depressed patients explain this finding. Other authors who also reported a strong correlation of symptoms with QoL did not find an effect of depression on QoL [21]. The negative effects of higher PASP and recent urgent HF visits on QoL were also captured in the final model. Diabetic patients constitute a subgroup of HF patients experiencing worse QoL, as reported by other authors [20,22]. Nevertheless, we did not verify a negative impact on QoL of other comorbidities, as found in a recent literature review [20].

We found some intriguing associations with QoL improvement and deterioration. Past smokers had better QoL, possibly reflecting healthier behaviour in patients who stopped smoking. Accordingly, we confirmed the negative impact of poor adherence to healthcare on QoL. Patients with LVEF between 41 and 49% at follow-up had worse QoL than patients with preserved LVEF. This is surprising, because an LVEF ≤ 40% at HF clinic admission was associated with better QoL in the univariate analysis, but its effect was captured in multivariate analyses. Patients with sleep-related disorders had better QoL, even though non-invasive ventilation had no impact on QoL. The negative impact of anaemia at clinic admission on QoL persisted throughout all the models. However, the negative effect of low haemoglobin at follow-up was captured in the final model, probably because patients with anaemia were classified with a higher NYHA class. The reasons behind these unexpected findings are not clear, but encourage further research.

In this study, age and HF aetiology had no effect on QoL. Patients with higher SBP and those taking an ACEi had significantly improved QoL. No impact was observed on QoL of the few patients taking sacubitril/valsartan or SGLT2i, contrary to the published evidence [23,24,25]. Clinical trials that measured the impact of ACE inhibitors on the QoL of patients with HF and reduced LVEF reported results that were confounded by a placebo effect, high noncompletion rates, different measurement tools, and evaluations of different QoL domains. Typically, QoL measurements in patients receiving ACEi showed small improvements or did not differ significantly from those in placebo-treated patients over long-term follow-up [26]. We found that higher furosemide doses and NT-proBNP levels are associated with worse QoL, as expected [21], since they are surrogate markers of severe HF.

Importantly, we found that NYHA class III and HFH were associated with large-to-very-large clinically important deterioration in QoL, whereas NYHA class II and an eGFR < 30 mL/min/1.73 m^2^ were associated with small-to-moderate clinically important QoL deterioration. Despite the association between higher NYHA classes and worse QoL, which is widely reflected in the literature [10,16,21], improving QoL does not always imply that NYHA class has improved. In fact, recently, finerenone improved QoL, but not NYHA class, in patients with LVEF ≥ 40% [27]. On the other hand, vericiguat was shown to reduce HFH in HF patients with LVEF < 45%, but had no impact on QoL [18]. Moreover, the most recent positive drug trials in HF patients revealed a non-clinically important QoL improvement, which was driven mainly by a reduction in HFH [10,28]. These observations underscore the relevance of looking at QoL improvement as a key therapeutic target. Furthermore, we found that improvement in one NYHA class, compared with worsening, had a more pronounced effect on QoL, which is in line with findings in other studies [9,29].

Patients who received a CRT-P showed a moderate-to-large clinically important improvement in QoL. Despite its well-established clinical benefits and cost-effectiveness, CRT-P remains widely underutilized. European data suggest that only one in three eligible patients receives a CRT device [30]. Zeitler et al. demonstrated that the burden of comorbidities does not appear to compromise the clinical benefits of CRT in terms of morbidity and mortality [31]. Indeed, an ESC position paper regarding CRT states that clinical factors favoring the use of CRT-P could include advanced age, more severe symptoms, and life-shortening comorbidities, such as stage IV CKD [30]. In fact, these are the characteristics of our patients in the current study.

As a result of our findings, consideration should be given to earlier CRT-P implantation in eligible patients, especially elderly patients with diabetes or a high burden of comorbidities. Overall, our study results should lead us to diagnose and treat HF sooner and aggressively, with the aim of maintaining patients in NYHA class I and free of HFH. If we achieve this high standard of HF care, we can not only reduce mortality, but also improve QoL.

Our study has limitations to acknowledge. This was a single-centre observational study. Thus, residual confounding cannot be ruled out, nor can the results be generalized. Additionally, the effects of obesity and iron replacement on QoL were not assessed. Nevertheless, we analysed a wide range of factors retrieved from patients, nurses, physicians, and laboratory and treatment data. The latter, combined with a well-characterized real-world sample of HF patients across the entire range of the LVEF spectrum, are major strengths of this study. Finally, we showed that it is possible to include QoL assessment in routine clinical practice.

## 5. Conclusions

In conclusion, this study revealed that patients who received a CRT-P had moderate-to-large clinically important improvements in QoL and that NYHA class II-IV, HFH and eGFR < 30 mL/min/1.73 m^2^ were associated with clinically important QoL deterioration. These were the only factors associated with clinically important QoL changes, considering a comprehensive number of factors studied and retrieved from patients, nurses, physicians, and laboratory and treatment data, while using a structured statistical analysis with nested LME models. These findings should lead clinicians to improve HF diagnosis and optimize treatment, including early CRT-P referrals for eligible patients. Consequently, clinically meaningful improvements in QoL may be expected. Future studies are needed to confirm our findings.

## Figures and Tables

**Table 1:** Baseline characteristics ^a^.

		*n*
Demographics ^b^		
Age (years), median (IQR)	78 (14)	419
Female, *n* (%)	228 (54.4)	419
Formal education (years), *n* (%)		363
0	52 (14.3)	
1–4	231 (63.6)	
>4	80 (22.0)	
Low income ^c^, *n* (%)	53 (12.6)	419
Comorbidities, *n* (%)		
Hypertension ^b^	357 (85.2)	419
Diabetes *mellitus*	225 (54.1)	416
Pre-diabetes	66 (15.8)	419
Dyslipidemia	318 (75.9)	419
Obesity ^b^	129 (30.9)	418
Smoking ^b^		419
Current	40 (9.5)	
Past	110 (26.3)	
Atrial flutter/fibrillation ^b,d^	247 (58.9)	419
Chronic kidney disease ^b^	230 (54.9)	419
Cerebrovascular disease	93 (22.2)	419
Peripheral arterial disease	58 (13.8)	419
Chronic pulmonary condition	156 (37.2)	419
Sleep-related breathing disorder	154 (36.8)	419
Anaemia ^b^	218 (52.2)	418
Iron deficiency	218 (71.5)	305
Cancer	14 (3.3)	419
Anxiety	25 (6.0)	419
Depression	124 (29.6)	419
Heart failure		
Etiology ^b^, *n* (%)		419
Hypertensive	178 (42.5)	
Ischemic	163 (38.9)	
Valvular	146 (34.8)	
NYHA functional class, *n* (%)		419
I	65 (15.5)	
II	262 (62.5)	
III	47 (11.2)	
IV	2 (0.5)	
SBP (mmHg), mean (SD)	126.7 (21.1)	399
Heart rate (bpm), mean (SD)	71.8 (12.1)	403
LVEF, *n* (%)		419
≥50%	216 (51.6)	
41–49%	40 (9.5)	
≤40%	163 (38.9)	
HF self-care, *n* (%)		
HF literacy	98 (25.7)	381
Self-care literacy	146 (37.5)	389
Self-care adherence	132 (33.9)	389
Exercice adherence	72 (20.7)	348
Poor adherence, *n* (%)	169 (40.3)	419
HF events ^e^, *n* (%)		419
HF hospitalization	39 (9.3)	
Urgent HF visit	4 (1.0)	
Laboratory		
Haemoglobin (g/dL), mean (SD)	12.5 (1.8)	419
Creatinine (mg/dL), mean (SD)	1.35 (0.64)	419
eGFR (mL/min/1.73 m^2^), *n* (%)		419
≥60	167 (39.9)	
45–59	78 (18.6)	
30–44	113 (27.0)	
<30	61 (14.6)	
NT-proBNP (pg/mL), median (IQR)	1454 (2546)	415
Medications, *n* (%)		419
ACEi	203 (48.4)	
ARNi	11 (2.6)	
ARB	41 (9.8)	
MRA	136 (32.5)	
BB	326 (77.8)	
SGLT2i	14 (3.3)	
Furosemide (mg/day), mean (SD)	80.0 (41.5)	
Other treatments, *n* (%)		
Cardiac rehabilitation	9 (2.1)	419
Nocturnal non-invasive ventilation	55 (13.1)	419
Cardiac devices	72 (17.2)	419
CRT-P	9 (2.1)	
CRT-D	8 (1.9)	
ICD	11 (2.6)	
Pacemaker	44 (10.5)	

ACEi, angiotensin-converting enzyme inhibitor; ARB, angiotensin-receptor blocker; ARNi, angiotensin receptor–neprilysin inhibitor; BB, beta-blocker; CRT-D, cardiac resynchronization therapy-defibrillator; CRT-P, cardiac resynchronization therapy-pacemaker; eGFR, estimated glomerular filtration rate; HF, heart failure; ICD, implantable cardioverter–defibrillator; IQR, interquartile range; LVEF, left ventricle ejection fraction; MRA, mineralocorticoid receptor antagonist; NT-proBNP, N-terminal-pro-B-type natriuretic peptide; NYHA, New York Heart Failure; SBP, systolic blood pressure; SD, standard deviation; SGLT2i, sodium–glucose co-transporter 2 inhibitor. ^a^ Baseline represents the moment of the first MLHFQ administration. ^b^ Data collected at HF clinic admission. ^c^ Self-reported. ^d^ Past or present history of atrial fibrillation or atrial flutter. ^e^ HF events on the previous 28 days.

**Table 2:** Mixed-effects models with MLHFQ total score, patient demographics and comorbidities ^a^.

Variable		Model 1			Model 2 ^b^			Model 3 ^c^			Model 4 ^d^			Model 5 ^e^	
		CI	*p*-Value		CI	*p*-Value		CI	*p*-Value		CI	*p*-Value		CI	*p*-Value
Age															
<65	Ref			Ref			Ref			Ref			Ref		
65–79	3.09	−1.25–7.42	0.162	2.82	−1.32–6.95	0.181	2.45	−1.78–6.68	0.255	2.37	−1.91–6.65	0.277	−0.09	−3.87–3.69	0.965
≥80	1.86	−2.57–6.28	0.409	2.26	−2.08–6.61	0.307	0.255	−2.08–6.86	0.294	2.20	−2.34–6.74	0.342	−2.15	−6.25–1.96	0.304
Sex															
Female	Ref			Ref			Ref			Ref			Ref		
Male	−4.69	−7.69–−1.68	**0.002**	−4.30	−7.30–−1.30	**0.005**	−4.46	−7.53–−1.39	**0.005**	−4.73	−7.85–−1.62	**0.003**	−1.50	−4.34–1.33	0.298
Low income															
No	Ref			Ref			Ref			Ref			Ref		
Yes	4.19	−0.43–8.81	0.076	4.67	0.08–9.25	**0.046**	4.39	−0.19–8.97	0.060	4.03	−0.61–8.67	0.089	3.74	−0.38–7.86	0.075
Diabetes *mellitus*															
No	Ref			Ref			Ref			Ref			Ref		
Yes	2.95	−0.09–5.99	0.057	2.28	−0.66–5.22	0.128	2.06	−0.92–5.04	0.175	2.25	−0.75–5.26	0.141	3.00	0.29–5.70	**0.030**
Smoking															
Never	Ref			Ref			Ref			Ref			Ref		
Past	−5.09	−8.53–−1.65	**0.004**	−2.90	−7.17–1.38	0.184	−5.01	−9.52–−0.51	**0.029**	−4.95	−9.51–−0.40	**0.033**	−4.36	−8.44–−0.27	**0.037**
Current	−2.20	−7.46–3.07	0.413	−0.43	−6.43–5.58	0.889	−3.30	−9.66–3.07	0.309	−3.15	−9.58–3.29	0.337	−2.52	−8.31–3.27	0.393
CKD															
No	Ref			Ref			Ref			Ref			Ref		
Yes	2.23	−0.84–5.30	0.154	2.38	−0.64–5.41	0.122	2.11	−0.92–5.15	0.172	1.95	−1.12–5.02	0.212	0.06	−2.72–2.85	0.963
Chronic pulmonary condition															
No	Ref			Ref			Ref			Ref			Ref		
Yes	2.06	−1.04–5.17	0.192	2.60	−0.47–5.67	0.097	3.02	−0.06–6.10	0.055	3.14	0.03–6.26	**0.048**	1.49	−1.33–4.32	0.300
Sleep-related breathing disorder															
No	Ref			Ref			Ref			Ref			Ref		
Yes	−0.86	−3.59–1.86	0.534	−0.74	−3.43–1.95	0.588	−0.77	−3.44–1.91	0.573	−0.68	−3.36–1.99	0.616	−2.84	−5.34–−0.35	**0.026**
Anxiety															
No	Ref			Ref			Ref			Ref			Ref		
Yes	7.05	0.73–13.37	**0.029**	6.33	0.20–12.46	**0.043**	5.96	−0.31–12.24	0.063	5.52	−0.87–11.90	0.090	4.36	−1.38–10.10	0.136
Depression															
No	Ref			Ref			Ref			Ref			Ref		
Yes	4.92	1.70–8.14	**0.003**	4.63	1.44–7.83	**0.005**	4.27	1.04–7.50	**0.010**	4.22	0.96–7.49	**0.011**	2.46	−0.53–5.46	0.107
Anaemia															
No	Ref			Ref			Ref			Ref			Ref		
Yes	3.32	0.26–6.38	**0.033**	3.49	0.54–6.43	**0.020**	3.75	0.78–6.72	**0.013**	3.72	0.72–6.73	**0.015**	2.75	0.05–5.46	**0.046**

CKD, chronic kidney disease; CI, 95% confidence interval; MLHFQ, Minnesota Living with Heart Failure Questionnaire. ^a^ All models were adjusted to time and time^2^. ^b^ All variables were adjusted to model 1, age, sex, and low income. ^c^ All variables were adjusted to model 2, CKD, anaemia, CPD, anxiety, and depression. ^d^ All variables were adjusted to model 3 and selfcare adherence. ^e^ All variables were adjusted to model 4, NYHA, and NT-proBNP.

**Table 3:** Mixed-effects models with MLHFQ total score, HF, laboratory and treatment variables ^a^.

Variable		Model 1			Model 2 ^b^			Model 3 ^c^			Model 4 ^d^			Model 5 ^e^	
		CI	*p*-Value		CI	*p*-Value		CI	*p*-Value		CI	*p*-Value		CI	*p*-Value
NYHA class															
I	Ref			Ref			Ref			Ref			Ref		
II	9.78	6.96–12.60	**<0.001**	10.58	7.64–13.51	**<0.001**	10.52	7.55–13.50	**<0.001**	10.20	7.20–13.19	**<0.001**	9.74	6.74–12.75	**<0.001**
III	29.94	25.67–34.22	**<0.001**	30.18	25.71–34.65	**<0.001**	29.53	25.01–34.05	**<0.001**	29.13	24.58–33.67	**<0.001**	28.39	23.82–32.96	**<0.001**
IV	59.48	33.04–85.93	**<0.001**	61.40	35.05–87.76	**<0.001**	61.89	35.58–88.21	**<0.001**	61.88	35.55–88.22	**<0.001**	60.59	34.46–86.72	**<0.001**
SBP	−0.06	0.12–0.00	0.052	−0.08	−0.14–−0.02	**0.010**	−0.08	−0.14–−0.02	**0.009**	−0.08	−0.15–−0.02	**0.008**	−0.06	−0.12–−0.00	**0.045**
LVEF															
≥50%	Ref			Ref			Ref			Ref			Ref		
41–49%	5.96	1.36–10.56	**0.011**	7.16	2.56–11.76	**0.002**	6.37	1.76–10.98	**0.007**	6.09	1.49–10.70	**0.010**	4.88	0.60–9.17	**0.026**
≤40%	−3.23	−6.18–−0.28	**0.032**	−2.12	−5.18–0.94	0.174	−1.68	−4.75–1.39	0.282	−1.68	−4.77–1.42	0.287	−1.78	−4.74–1.19	0.239
PASP	0.24	0.12–0.36	**<0.001**	0.23	0.11–0.35	**<0.001**	0.20	0.08–0.32	**0.001**	0.20	0.08–0.32	**0.001**	0.11	−0.00–0.23	0.057
HF literacy															
No	Ref			Ref			Ref			Ref			Ref		
Yes	−2.97	−5.90–−0.03	**0.047**	−2.82	−5.73–0.08	0.057	−2.95	−5.86–−0.03	**0.047**	−2.24	−5.17–0.70	0.135	−2.50	−5.26–0.25	0.074
Self-care literacy															
No	Ref			Ref			Ref			Ref			Ref		
Yes	−4.47	−7.29–−1.65	**0.002**	−4.07	−6.91–−1.23	**0.005**	−3.90	−6.74–−1.07	**0.007**	−0.76	−4.83–3.30	0.713	−1.07	−5.04–2.91	0.599
Self-care adherence															
No	Ref			Ref			Ref			Ref			Ref		
Yes	−4.75	−7.28–−2.23	**<0.001**	−4.50	−7.05–−1.95	**0.001**	−4.33	−6.87–−1.79	**0.001**	−4.33	−6.87–−1.79	**0.001**	−2.26	−4.68–0.17	0.069
Exercise adherence															
No	Ref			Ref			Ref			Ref			Ref		
Yes	−3.43	−6.24–−0.62	**0.017**	−3.48	−6.32–−0.65	**0.016**	−2.99	−5.83–−0.14	**0.040**	−2.03	−4.99–0.92	0.177	−2.12	−4.87–0.63	0.130
Poor adherence															
No	Ref			Ref			Ref			Ref			Ref		
Yes	3.31	0.18–6.43	**0.038**	3.23	0.13–6.32	**0.041**	3.22	0.12–6.32	**0.042**	2.79	−0.37–5.95	0.083	3.36	0.53–6.20	**0.020**
HF hospitalization ^f^															
**No**	Ref			Ref			Ref			Ref			Ref		
Yes	32.75	27.46–38.03	**<0.001**	32.66	27.38–37.93	**<0.001**	32.00	26.74–37.26	**<0.001**	31.21	25.95–36.46	**<0.001**	26.91	21.80–32.03	**<0.001**
Urgent HF visit ^f^															
No	Ref			Ref			Ref			Ref			Ref		
Yes	15.25	3.78–26.71	**0.009**	17.05	6.44–27.67	**0.002**	16.37	5.75–26.99	**0.003**	16.47	5.83–27.10	**0.002**	8.79	−0.99–18.57	0.078
Haemoglobin ^g^															
Normal	Ref			Ref			Ref			Ref			Ref		
Low	4.46	1.92–7.00	**0.001**	4.59	2.04–7.15	**<0.001**	4.24	1.47–7.01	**0.003**	4.44	1.68–7.20	**0.002**	2.37	−0.27–5.00	0.078
eGFR															
≥60	Ref			Ref			Ref			Ref			Ref		
45–59	0.39	−2.90–3.68	0.816	0.50	−2.88–3.89	0.770	0.40	−3.17–3.97	0.825	0.46	−3.09–4.02	0.798	1.07	−2.25–4.39	0.529
30–44	2.14	−1.25–5.52	0.215	2.04	−1.43–5.50	0.249	1.76	−2.20–5.72	0.383	1.73	−2.22–5.68	0.390	−0.33	−4.04–3.37	0.860
<30	9.77	5.53–14.02	**<0.001**	9.33	5.06–13.60	**<0.001**	9.44	4.57–14.32	**<0.001**	9.60	4.73–14.47	**<0.001**	5.82	1.17–10.47	**0.014**
NT-proBNP/1000	0.37	0.20–0.54	**<0.001**	0.37	0.20–0.54	**<0.001**	0.37	0.20–0.54	**<0.001**	0.35	0.18–0.52	**<0.001**	0.20	0.03–0.36	**0.023**
ACEi															
No	Ref			Ref			Ref			Ref			Ref		
Yes	−4.67	−7.37–−1.96	**0.001**	−4.79	−7.50–−2.08	**0.001**	−4.20	−6.93–−1.48	**0.003**	−3.83	−6.58–−1.07	**0.007**	−3.45	−6.00–−0.91	**0.008**
Furosemide dose/40	3.78	2.64–4.92	**<0.001**	3.95	2.82–5.07	**<0.001**	3.80	2.65–4.94	**<0.001**	3.76	2.61–4.91	**<0.001**	2.29	1.09–3.49	**<0.001**
Cardiac rehabilitation															
No	Ref			Ref			Ref			Ref			Ref		
Yes	−5.82	−9.70–−1.94	**0.003**	−6.17	−10.02–−2.32	**0.002**	−5.92	−9.80–−2.05	**0.003**	−5.38	−9.24–−1.52	**0.006**	−2.90	−6.48–0.67	0.112
Cardiac device															
None	Ref			Ref			Ref			Ref			Ref		
ICD	−6.69	−14.36–0.98	0.087	−4.37	−12.17–3.42	0.271	−2.83	−10.59–4.93	0.474	−3.84	−11.61–3.93	0.332	−5.29	−12.34–1.77	0.142
CRT-P	−10.39	−19.71–−1.08	**0.029**	−9.49	−18.63–−0.35	**0.042**	−8.56	−17.61–0.50	0.064	−9.44	−18.41–−0.47	**0.039**	−13.55	−22.45–−4.65	**0.003**
CRT-D	0.03	−8.39–8.45	0.995	1.34	−6.76–9.44	0.745	−0.09	−8.20–8.02	0.982	−0.28	−8.32–7.76	0.945	0.61	−6.75–7.97	0.872
Pacemaker	−0.20	−4.65–4.26	0.931	0.30	−4.07–4.67	0.892	−0.19	−4.57–4.18	0.931	−0.65	−5.03–3.74	0.772	−1.88	−5.87–2.11	0.354

ACEi, angiotensin-converting enzyme inhibitor; CI, 95% confidence interval; CRT-D, cardiac resynchronization therapy-defibrillator; CRT-P, cardiac resynchronization therapy-pacemaker; eGFR, estimated glomerular filtration rate; HF, heart failure; ICD, implantable cardioverter–defibrillator; LVEF, left ventricle ejection fraction; MLHFQ, Minnesota Living with Heart Failure Questionnaire; NT-proBNP, N-terminal-pro-B type natriuretic peptide; NYHA, New York Heart Failure; PASP, pulmonary artery systolic pressure; RV, right ventricle; SBP, systolic blood pressure. ^a^ All models were adjusted to time and time^2^. ^b^ All variables were adjusted to model 1, age, sex, and low income. ^c^ All variables were adjusted to model 2, CKD, anaemia, CPD, anxiety, and depression. ^d^ All variables were adjusted to model 3 and selfcare adherence. ^e^ All variables were adjusted to model 4, NYHA, and NT-proBNP. ^f^ HF events on the previous 28 days. ^g^ Normal haemoglobin is defined as ≥12 g/dL in female and ≥13 g/dL in male patients.

## Data Availability

The raw data supporting the conclusions of this article will be made available by the authors on request.

## Supplementary Materials

The following supporting information can be downloaded at: https://www.mdpi.com/article/10.3390/jcm14145079/s1, Table S1: Patients’ characteristics at HF clinic admission and at baseline; Table S2: Mixed-effects models with MLHFQ total score, patient’s demographics and comorbidities; Table S3: Mixed-effects models with MLHFQ total score, HF and laboratory variables; Table S4: Mixed-effects models with MLHFQ total score and treatments.

## Abbreviations

The following abbreviations are used in this manuscript:

ACEi	Angiotensin-converting enzyme inhibitor
ARB	Angiotensin-receptor blocker
ARNi	Angiotensin receptor and neprilysin inhibitor
BB	Beta-blockers
CI	95% confidence interval
CKD	Chronic kidney disease
CKD-EPI	Chronic Kidney Disease Epidemiology Collaboration
CRT-D	Cardiac resynchronization-therapy defibrillator
CRT-P	Cardiac resynchronization-therapy pacemaker
DOAC	Direct-acting oral anticoagulant
eGFR	Estimated glomerular filtration rate
HF	Heart failure
HFH	Hearf failure hospitalization
ICD	Implantable cardioverter–defibrillator
IQR	Interquartile range
LME	Linear mixed effects
LVEF	Left ventricle ejection fraction
MLHFQ	Minnesota Living with Heart Failure Questionnaire
MRA	Mineralocorticoid receptor antagonists
NT-proBNP	N-terminal-pro-B type natriuretic peptide
NYHA	New York Heart Failure
PASP	Pulmonary artery systolic pressure
QoL	Quality of life
QUALIFIER	QUALIty oF lIfe in hEart failuRe
RAASi	Renin–angiotensin–aldosterone system inhibitor
RV	Right ventricle
SBP	Systolic blood pressure
SD	Standard deviation
SGLT2i	Sodium–glucose cotransporter 2 inhibitor
