# Supplementary material for: Factors Associated with Clinically Important Changes in Quality of Life of Heart Failure Patients: The QUALIFIER Prospective Cohort Study
Source: J Clin Med. 2025 Jul 17;14(14):5079. doi: 10.3390/jcm14145079 (PMC12295941; doi:10.3390/jcm14145079)
Supplement: Supplementary file 1 [file jcm-14-05079-s001.zip › Table S1.pdf]

Table S1. Patients´ characteristics at HF clinic admission and at baseline [a]

Variable	HF clinic admission		Baseline[a]	
Demographics		n		n
Age (years), median (IQR)	78 (14)	419		
Female, n (%)	228 (54.4)	419		
Formal education (years), n (%)		363		
0	52 (14.3)			
1-4	231 (63.6)			
> 4	80 (22.0)			
Low income [b], n (%)	53 (12.6)	419		
Comorbidities, n (%)				
Hypertension	357 (85.2)	419		
Diabetes *mellitus*	224 (53.8)	416	225 (54.1)	416
Pre-diabetes	58 (13.8)	419	66 (15.8)	419
Dyslipidemia	314 (74.9)	419	318 (75.9)	419
Obesity	129 (30.9)	418		
Smoking		419		
Current	40 (9.5)			
Past	110 (26.3)			
Atrial flutter/fibrillation [c]	247 (58.9)	419		
CKD	230 (54.9)	419		
Cerebrovascular disease	93 (22.2)	419	93 (22.2)	419
Peripheral arterial disease	58 (13.8)	419	58 (13.8)	419
Chronic pulmonary condition	153 (36.5)	419	156 (37.2)	419
Sleep-related breathing disorder	72 (17.2)	419	154 (36.8)	419
Anaemia	218 (52.2)	418		
Iron deficiency	175 (63.4)	276	218 (71.5)	305
Cancer	12 (2.9)	419	14 (3.3)	419
Sexual dysfunction	17 (4.1)	419	17 (4.1)	419
Anxiety	25 (6.0)	419	25 (6.0)	419
Depression	121 (28.9)	419	124 (29.6)	419
Heart failure				
Etiology, n (%)		419		
Hypertensive	178 (42.5)			
Ischemic	163 (38.9)			
Valvular	146 (34.8)			
Dilated non-ischemic	32 (7.6)			
NYHA functional class, n (%)		419		419
I	58 (13.8)		65 (15.5)	
II	269 (64.2)		262 (62.5)	
III	70 (16.7)		47 (11.2)	
IV	4 (1.0)		2 (0.5)	
Not classified	18 (4.3)		43 (10.3)	
SBP (mmHg), mean (SD)			126.7 (21.1)	399
Heart rate (bpm), mean (SD)			71.8 (12.1)	403
Rhythm, n (%)		419		419

Sinus	227 (54.2)		225 (53.7)	
Atrial flutter/fibrillation	167 (39.9)		152 (36.3)	
Pacemaker	25 (6.0)		42 (10.0)	
LVEF, n (%)		419		419
≥50%	208 (49.6)		216 (51.6)	
41-49%	41 (9.8)		40 (9.5)	
≤40%	170 (40.6)		163 (38.9)	
RV systolic dysfunction, n (%)			72 (17.3)	415
PASP (mmHg), mean (SD)			40 (13)	315
Severe tricuspide regurgitation, n (%)			14 (3.4)	416
HF self-care, n (%)				
HF literacy			98 (25.7)	381
Self-care literacy			146 (37.5)	389
Self-care adherence			132 (33.9)	389
Exercice adherence			72 (20.7)	348
Poor adherence, n (%)			169 (40.3)	419
HF events [d], n (%)				419
HF hospitalization			39 (9.3)	
Urgent HF visit			4 (1.0)	
Laboratory				
Haemoglobin (g/dL), mean (SD)	12.3 (1.8)	418	12.5 (1.8)	419
Creatinine (mg/dL), mean (SD)	1.27 (0.62)	419	1.35 (0.64)	419
eGFR (mL/min/1.73 m^2), n (%)		419		419
≥60	175 (41.8)		167 (39.9)	
45-59	96 (22.9)		78 (18.6)	
30-44	106 (25.3)		113 (27.0)	
<30	42 (10.0)		61 (14.6)	
NT-proBNP (pg/mL), mean (SD)	2423 (4094)	383	1454 (2546)	415
Medications, n (%)				419
ACEi			203 (48.4)	
ARNi			11 (2.6)	
ARB			41 (9.8)	
MRA			136 (32.5)	
BB			326 (77.8)	
SGLT2i			14 (3.3)	
Ivabradine			13 (3.1)	
Furosemide (mg/day), mean (SD)			80.0 (41.5)	
Anticoagulants			218 (52.0)	
Other treatments, n (%)				
Cardiac rehabilitation			9 (2.1)	419
Nocturnal non-invasive ventilation			55 (13.1)	419
Cardiac devices			72 (17.2)	419
CRT-P			9 (2.1)	
CRT-D			8 (1.9)	
ICD			11 (2.6)	
Pacemaker			44 (10.5)	

ACEi, angiotensin-converting enzyme inhibitor; ARB, angiotensin-receptor blocker; ARNi, angiotensin receptor-neprilysin inhibitor; BB, beta-blocker; CKD, chronic kidney disease; CRT-D, cardiac resynchronization therapy-defibrillator; CRT-P, cardiac resynchronization therapy-pacemaker; eGFR, estimated glomerular filtration rate; HF, heart failure; ICD, implantable cardioverter-defibrillator; IQR, interquartile range; LVEF, left ventricle ejection fraction; MRA, mineralocorticoid receptor antagonist; NT-proBNP, N-terminal-pro-B type natriuretic peptide; NYHA, New York Heart Failure; PASP, pulmonary artery systolic pressure; RV, right ventricle; SBP, systolic blood pressure; SD, standard deviation; SGLT2i, sodium-glucose co-transporter 2 inhibitor. [a] Baseline represents the moment of the first MLHFQ administration. [b] Self-reported. [c] Past or present history of atrial fibrillation or atrial flutter. [d] HF events on the previous 28 days.
