# Supplementary material for: Factors Associated with Clinically Important Changes in Quality of Life of Heart Failure Patients: The QUALIFIER Prospective Cohort Study
Source: J Clin Med. 2025 Jul 17;14(14):5079. doi: 10.3390/jcm14145079 (PMC12295941; doi:10.3390/jcm14145079)
Supplement: Supplementary file 1 [file jcm-14-05079-s001.zip › Table S2.pdf]

Table S2. Mixed effects models with MLHFQ total score, patient's demographics and comorbidities [a]

Variable		Model 1			Model 2 [b]			Model 3 [c]			Model 4 [d]			Model 5 [e]	
		CI	*p*-value		CI	*p*-value		CI	*p*-value		CI	*p*-value		CI	*p*-value
Age															
< 65	Ref			Ref			Ref			Ref			Ref		
65-79	3.09	-1.25 – 7.42	0.162	2.82	-1.32 – 6.95	0.181	2.45	-1.78 – 6.68	0.255	2.37	-1.91 – 6.65	0.277	-0.09	-3.87 – 3.69	0.965
≥ 80	1.86	-2.57 – 6.28	0.409	2.26	-2.08 – 6.61	0.307	0.255	-2.08 – 6.86	0.294	2.20	-2.34 – 6.74	0.342	-2.15	-6.25 – 1.96	0.304
Sex															
Female	Ref			Ref			Ref			Ref			Ref		
Male	-4.69	-7.69 – -1.68	**0.002**	-4.30	-7.30 – -1.30	**0.005**	-4.46	-7.53 – -1.39	**0.005**	-4.73	-7.85 – -1.62	**0.003**	-1.50	-4.34 – 1.33	0.298
Formal education															
0	Ref														
1-4	-1.50	-6.67 – 3.66	0.568												
>4	-2.76	-8.64 – 3.12	0.357												
Low income															
No	Ref			Ref			Ref			Ref			Ref		
Yes	4.19	-0.43 - 8.81	0.076	4.67	0.08 – 9.25	**0.046**	4.39	-0.19 – 8.97	0.060	4.03	-0.61 – 8.67	0.089	3.74	-0.38 – 7.86	0.075
Hypertension															
No	Ref			Ref			Ref			Ref			Ref		
Yes	0.50	-3.73 - 4.74	0.815	-0.50	-4.57 – 3.56	0.807	-0.12	-4.21 – 3.97	0.953	-0.20	-4.33 – 3.93	0.924	0.04	-3.61 – 3.70	0.981
Diabetes *mellitus*															
No	Ref			Ref			Ref			Ref			Ref		
Yes	2.95	-0.09 - 5.99	0.057	2.28	-0.66 – 5.22	0.128	2.06	-0.92 – 5.04	0.175	2.25	-0.75 – 5.26	0.141	3.00	0.29 – 5.70	**0.030**
Pre-diabetes															
No	Ref			Ref			Ref			Ref			Ref		
Yes	-2.34	-6.08 - 1.41	0.221	-2.21	-5.84 – 1.41	0.230	-2.01	-5.65 – 1.62	0.277	-2.04	-5.70 – 1.61	0.273	-1.46	-4.79 – 1.88	0.391
Smoking															
Never	Ref			Ref			Ref			Ref			Ref		

Past	-5.09	-8.53 – -1.65	**0.004**	-2.90	-7.17 – 1.38	0.184	-5.01	-9.52 – -0.51	**0.029**	-4.95	-9.51 – -0.40	**0.033**	-4.36	-8.44 – -0.27	**0.037**
Current	-2.20	-7.46 – 3.07	0.413	-0.43	-6.43 – 5.58	0.889	-3.30	-9.66 – 3.07	0.309	-3.15	-9.58 – 3.29	0.337	-2.52	-8.31 – 3.27	0.393
CKD															
No	Ref			Ref			Ref			Ref			Ref		
Yes	2.23	-0.84 - 5.30	0.154	2.38	-0.64 – 5.41	0.122	2.11	-0.92 – 5.15	0.172	1.95	-1.12 – 5.02	0.212	0.06	-2.72 – 2.85	0.963
Cerebrovascular disease															
No	Ref			Ref			Ref			Ref			Ref		
Yes	0.32	-3.13 - 3.76	0.857	0.34	-3.02 – 3.70	0.843	0.04	-3.31 – 3.38	0.984	0.41	-2.96 – 3.78	0.812	-0.36	-3.45 – 2.73	0.819
Peripheral arterial disease															
No	Ref			Ref			Ref			Ref			Ref		
Yes	2.22	-2.09 - 6.53	0.313	2.71	-1.55 – 6.98	0.212	2.03	-2.25 – 6.30	0.352	1.71	-2.62 – 6.04	0.438	-1.74	-5.74 – 2.25	0.392
Chronic pulmonary condition															
No	Ref			Ref			Ref			Ref			Ref		
Yes	2.06	-1.04 - 5.17	0.192	2.60	-0.47 – 5.67	0.097	3.02	-0.06 – 6.10	0.055	3.14	0.03 – 6.26	**0.048**	1.49	-1.33 – 4.32	0.300
Sleep-related breathing disorder															
No	Ref			Ref			Ref			Ref			Ref		
Yes	-0.86	-3.59 - 1.86	0.534	-0.74	-3.43 – 1.95	0.588	-0.77	-3.44 – 1.91	0.573	-0.68	-3.36 – 1.99	0.616	-2.84	-5.34 – -0.35	**0.026**
Anaemia															
No	Ref			Ref			Ref			Ref			Ref		
Yes	3.32	0.26 - 6.38	**0.033**	3.49	0.54 – 6.43	**0.020**	3.75	0.78 – 6.72	**0.013**	3.72	0.72 – 6.73	**0.015**	2.75	0.05 – 5.46	**0.046**
Iron deficiency															
No	Ref			Ref			Ref			Ref			Ref		
Yes	3.86	-0.05 - 7.78	0.053	2.46	-1.55 – 6.47	0.229	2.06	-1.98 – 6.09	0.317	2.46	-1.60 – 6.53	0.234	1.40	-2.40 – 5.19	0.471
Unknown	-0.36	-4.91 - 4.19	0.876	-0.53	-5.00 – 3.93	0.814	0.52	-3.98 – 5.02	0.819	0.88	-3.65 – 5.42	0.702	2.46	-1.68 – 6.61	0.243
Cancer															
No	Ref			Ref			Ref			Ref			Ref		
Yes	4.87	-2.57 - 12.31	0.199	5.83	-1.39 – 13.05	0.114	4.88	-2.34 – 12.11	0.185	4.23	-2.97 – 11.43	0.249	3.37	-3.16 – 9.91	0.310
Sexual dysfunction															
No	Ref			Ref			Ref			Ref			Ref		
Yes	-0.82	-8.37 - 6.73	0.832	1.11	-6.39 – 8.61	0.772	2.41	-5.06 – 9.87	0.527	2.84	-4.67 – 10.35	0.458	2.79	-3.86 – 9.44	0.410

Anxiety															
No	Ref			Ref			Ref			Ref			Ref		
Yes	7.05	0.73 - 13.37	**0.029**	6.33	0.20 – 12.46	**0.043**	5.96	-0.31 – 12.24	0.063	5.52	-0.87 – 11.90	0.090	4.36	-1.38 – 10.10	0.136
Depression															
No	Ref			Ref			Ref			Ref			Ref		
Yes	4.92	1.70 - 8.14	**0.003**	4.63	1.44 – 7.83	**0.005**	4.27	1.04 – 7.50	**0.010**	4.22	0.96 – 7.49	**0.011**	2.46	-0.53 – 5.46	0.107

CI, 95% confidence interval; CKD, chronic kidney disease; MLHFQ, Minnesota Living with Heart Failure Questionnaire. [a] All models were adjusted to time and time^2. [b] All variables were adjusted to model 1, age, sex, and low income. [c] All variables were adjusted to model 2, CKD, anaemia, CPD, anxiety, and depression. [d] All variables were adjusted to model 3 and selfcare adherence. [e] All variables were adjusted to model 4, NYHA, and NT-proBNP.
