# Supplementary material for: Factors Associated with Clinically Important Changes in Quality of Life of Heart Failure Patients: The QUALIFIER Prospective Cohort Study
Source: J Clin Med. 2025 Jul 17;14(14):5079. doi: 10.3390/jcm14145079 (PMC12295941; doi:10.3390/jcm14145079)
Supplement: Supplementary file 1 [file jcm-14-05079-s001.zip › Table S3.pdf]

Table S3.Mixed effects models with MLHFQ total score, HF and laboratory variables [a]

**Variable**	**Model 1**			**Model 2 [b]**			**Model 3 [c]**			**Model 4 [d]**			**Model 5 [e]**		
		CI	*p*-value		CI	*p*-value		CI	*p*-value		CI	*p*-value		CI	*p*-value
Ischemic etiology															
No	Ref			Ref			Ref			Ref			Ref		
Yes	0.81	-2.33 - 3.94	0.613	2.19	-0.95 – 5.33	0.172	1.29	-1.89 – 4.46	0.427	1.18	-2.03 – 4.40	0.470	0.41	-2.46 – 3.27	0.781
Hypertensive etiology															
No	Ref			Ref			Ref			Ref			Ref		
Yes	-0.40	-3.50 – 2.71	0.802	-1.09	-4.16 – 1.98	0.485	-0.47	-3.56 – 2.61	0.763	-0.32	-3.44 – 2.80	0.841	0.49	-2.30 – 3.29	0.728
Valvular etiology															
No	Ref			Ref			Ref			Ref			Ref		
Yes	3.46	0.24 – 6.68	**0.035**	2.66	-0.54 – 5.87	0.103	2.21	-0.99 – 5.41	0.175	2.21	-1.03 – 5.44	0.181	-0.07	-3.00 – 2.87	0.965
Nonischemic dilated etiology															
No	Ref			Ref			Ref			Ref			Ref		
Yes	-4.20	-9.60 - 1.21	0.128	-2.94	-8.34 – 2.46	0.286	-2.28	-7.73 – 3.16	0.410	-2.25	-7.78 – 3.29	0.426	-2.11	-6.83 – 2.60	0.379
NYHA class															
I	Ref			Ref			Ref			Ref			Ref		
II	9.78	6.96 - 12.60	**<0.001**	10.58	7.64 – 13.51	**<0.001**	10.52	7.55 – 13.50	**<0.001**	10.20	7.20 – 13.19	**<0.001**	9.74	6.74 – 12.75	**<0.001**
III	29.94	25.67 - 34.22	**<0.001**	30.18	25.71 – 34.65	**<0.001**	29.53	25.01 – 34.05	**<0.001**	29.13	24.58 – 33.67	**<0.001**	28.39	23.82 – 32.96	**<0.001**
IV	59.48	33.04 - 85.93	**<0.001**	61.40	35.05 – 87.76	**<0.001**	61.89	35.58 – 88.21	**<0.001**	61.88	35.55 – 88.22	**<0.001**	60.59	34.46 – 86.72	**<0.001**
SBP	-0.06	0.12 - 0.00	0.052	-0.08	-0.14 – -0.02	**0.010**	-0.08	-0.14 – -0.02	**0.009**	-0.08	-0.15 – -0.02	**0.008**	-0.06	-0.12 – -0.00	**0.045**
Heart rate	0.03	-0.07 - 0.13	0.602	0.02	-0.08 – 0.12	0.696	0.03	-0.07 – 0.13	0.603	0.02	-0.08 – 0.12	0.677	0.09	-0.01 – 0.19	0.069
Rhythm															
Sinus	Ref			Ref			Ref			Ref			Ref		
Atrial fibrillation	-0.35	-3.42 - 2.72	0.823	-0.06	-3.17 – 3.05	0.971	0.57	-2.54 – 3.67	0.721	0.93	-2.20 – 4.05	0.561	-0.49	-3.40 – 2.41	0.739
Pacemaker	1.28	-3.43 - 6.00	0.593	2.18	-2.49 – 6.86	0.360	1.94	-2.71 – 6.58	0.413	1.24	-3.41 – 5.88	0.601	-0.50	-4.87 – 3.87	0.822
LVEF															
≥ 50%	Ref			Ref			Ref			Ref			Ref		

41-49%	5.96	1.36 – 10.56	**0.011**	7.16	2.56 – 11.76	**0.002**	6.37	1.76 – 10.98	**0.007**	6.09	1.49 – 10.70	**0.010**	4.88	0.60 – 9.17	**0.026**
≤ 40%	-3.23	-6.18 – -0.28	**0.032**	-2.12	-5.18 – 0.94	0.174	-1.68	-4.75 – 1.39	0.282	-1.68	-4.77 – 1.42	0.287	-1.78	-4.74 – 1.19	0.239
RV systolic dysfunction															
No	Ref			Ref			Ref			Ref			Ref		
Yes	2.71	-1.12 - 6.53	0.166	3.71	-0.11 – 7.54	0.057	3.77	-0.02 – 7.57	0.051	3.65	-0.15 – 7.44	0.060	2.71	-0.88 – 6.30	0.138
PASP	0.24	0.12 - 0.36	**<0.001**	0.23	0.11 – 0.35	**<0.001**	0.20	0.08 – 0.32	**0.001**	0.20	0.08 – 0.32	**0.001**	0.11	-0.00 – 0.23	0.057
Severe Tricuspid regurgitation															
No	Ref			Ref			Ref			Ref			Ref		
Yes	4.24	4.54 -13.02	0.343	3.86	-4.78 – 12.50	0.380	2.53	-6.05 – 11.11	0.562	3.62	-4.96 – 12.19	0.408	1.70	-6.48 – 9.88	0.684
HF literacy															
No	Ref			Ref			Ref			Ref			Ref		
Yes	-2.97	-5.90 – -0.03	**0.047**	-2.82	-5.73 – 0.08	0.057	-2.95	-5.86 – -0.03	**0.047**	-2.24	-5.17 – 0.70	0.135	-2.50	-5.26 – 0.25	0.074
Self-care literacy															
No	Ref			Ref			Ref			Ref			Ref		
Yes	-4.47	-7.29 – -1.65	**0.002**	-4.07	-6.91 – -1.23	**0.005**	-3.90	-6.74 – -1.07	**0.007**	-0.76	-4.83 – 3.30	0.713	-1.07	-5.04 – 2.91	0.599
Self-care adherence															
No	Ref			Ref			Ref			Ref			Ref		
Yes	-4.75	-7.28 – -2.23	**<0.001**	-4.50	-7.05 – -1.95	**0.001**	-4.33	-6.87 – -1.79	**0.001**	-4.33	-6.87 – -1.79	**0.001**	-2.26	-4.68 – 0.17	0.069
Exercice adherence															
No	Ref			Ref			Ref			Ref			Ref		
Yes	-3.43	-6.24 – -0.62	**0.017**	-3.48	-6.32 – -0.65	**0.016**	-2.99	-5.83 – -0.14	**0.040**	-2.03	-4.99 – 0.92	0.177	-2.12	-4.87 – 0.63	0.130
Poor adherence															
No	Ref			Ref			Ref			Ref			Ref		
Yes	3.31	0.18 - 6.43	**0.038**	3.23	0.13 – 6.32	**0.041**	3.22	0.12 – 6.32	**0.042**	2.79	-0.37 – 5.95	0.083	3.36	0.53 – 6.20	**0.020**
HF hospitalization[f]															
No	Ref			Ref			Ref			Ref			Ref		
Yes	32.75	27.46 – 38.03	**<0.001**	32.66	27.38 – 37.93	**<0.001**	32.00	26.74 – 37.26	**<0.001**	31.21	25.95 – 36.46	**<0.001**	26.91	21.80 – 32.03	**<0.001**
Urgent HF visit[f]															
No	Ref			Ref			Ref			Ref			Ref		

Yes	15.25	3.78 – 26.71	**0.009**	17.05	6.44 – 27.67	**0.002**	16.37	5.75 – 26.99	**0.003**	16.47	5.83 – 27.10	**0.002**	8.79	-0.99 – 18.57	0.078
Haemoglobin[g]															
Normal	Ref			Ref			Ref			Ref			Ref		
Low	4.46	1.92 -7.00	**0.001**	4.59	2.04 – 7.15	**<0.001**	4.24	1.47 – 7.01	**0.003**	4.44	1.68 – 7.20	**0.002**	2.37	-0.27 – 5.00	0.078
eGFR															
≥ 60	Ref			Ref			Ref			Ref			Ref		
45-59	0.39	-2.90 – 3.68	0.816	0.50	-2.88 – 3.89	0.770	0.40	-3.17 – 3.97	0.825	0.46	-3.09 – 4.02	0.798	1.07	-2.25 – 4.39	0.529
30-44	2.14	-1.25 – 5.52	0.215	2.04	-1.43 – 5.50	0.249	1.76	-2.20 – 5.72	0.383	1.73	-2.22 – 5.68	0.390	-0.33	-4.04 – 3.37	0.860
< 30	9.77	5.53 – 14.02	**<0.001**	9.33	5.06 – 13.60	**<0.001**	9.44	4.57 – 14.32	**<0.001**	9.60	4.73 – 14.47	**<0.001**	5.82	1.17 – 10.47	**0.014**
NT-proBNP/1000	0.37	0.20 - 0.54	**<0.001**	0.37	0.20 – 0.54	**<0.001**	0.37	0.20 – 0.54	**<0.001**	0.35	0.18 – 0.52	**<0.001**	0.20	0.03 – 0.36	**0.023**

CI, 95% confidence interval; eGFR, estimated glomerular filtration rate; HF, heart failure; LVEF, left ventricle ejection fraction; MLHFQ, Minnesota Living with Heart Failure Questionnaire, NT-proBNP, N-terminal-pro-B type natriuretic peptide; NYHA, New York Heart Failure; PASP, pulmonary artery systolic pressure; RV, right ventricle; SBP, systolic blood pressure. [a] All models were adjusted to time and time^2. [b] All variables were adjusted to model 1, age, sex, and low income. [c] All variables were adjusted to model 2, CKD, anaemia, CPD, anxiety, and depression. [d] All variables were adjusted to model 3 and selfcare adherence. [e] All variables were adjusted to model 4, NYHA, and NT-proBNP. [f] HF events on the previous 28 days. [g] Normal haemoglobin is defined as ≥12 g/dL in female and ≥13 g/dL in male patients.
