# Supplementary material for: Factors Associated with Clinically Important Changes in Quality of Life of Heart Failure Patients: The QUALIFIER Prospective Cohort Study
Source: J Clin Med. 2025 Jul 17;14(14):5079. doi: 10.3390/jcm14145079 (PMC12295941; doi:10.3390/jcm14145079)
Supplement: Supplementary file 1 [file jcm-14-05079-s001.zip › Table S4.pdf]

Table S4. Mixed effects models with MLHFQ total score and treatments [a]

**Variable**	**Model 1**			**Model 2 [b]**			**Model 3 [c]**			**Model 4 [d]**			**Model 5 [e]**		
		CI	*p*-value		CI	*p*-value		CI	*p*-value		CI	*p*-value		CI	*p*-value
ACEi															
No	Ref			Ref			Ref			Ref			Ref		
Yes	-4.67	-7.37 – -1.96	**0.001**	-4.79	-7.50 – -2.08	**0.001**	-4.20	-6.93 – -1.48	**0.003**	-3.83	-6.58 – -1.07	**0.007**	-3.45	-6.00 – -0.91	**0.008**
ARNi															
No	Ref			Ref			Ref			Ref			Ref		
Yes	-1.24	-8.17 – 5.69	0.726	-0.25	-7.17 – 6.68	0.944	-0.01	-6.93 – 6.90	0.997	-0.60	-7.54 – 6.35	0.866	0.07	-6.34 – 6.49	0.982
ARB															
No	Ref			Ref			Ref			Ref			Ref		
Yes	1.50	-3.63 – 6.63	0.566	0.76	-4.31 – 5.82	0.770	1.37	-3.68 – 6.41	0.595	1.32	-3.78 – 6.42	0.611	4.29	-0.22 – 8.80	0.062
MRA															
No	Ref			Ref			Ref			Ref			Ref		
Yes	-0.50	-3.51 – 2.50	0.742	0.12	-2.86 – 3.09	0.938	1.00	-2.00 – 3.99	0.514	1.04	-1.96 – 4.04	0.495	-0.02	-2.84 – 2.79	0.987
BB															
No	Ref			Ref			Ref			Ref			Ref		
Yes	-1.63	-4.85 - 1.58	0.319	-0.83	-4.08 – 2.42	0.616	-0.11	-3.36 – 3.13	0.946	-0.17	-3.42 – 3.08	0.919	0.87	-2.18 – 3.92	0.575
SGLT2i															
No	Ref			Ref			Ref			Ref			Ref		
Yes	-3.91	-9.54 - 1.73	0.174	-4.65	-10.21 – 0.91	0.101	-4.74	-10.27 – 0.80	0.094	-5.22	-10.70 – 0.26	0.062	-1.94	-7.14 – 3.25	0.462
Ivabradine															
No	Ref			Ref			Ref			Ref			Ref		
Yes	-1.48	-8.18 – 5.22	0.665	0.07	-6.53 – 6.66	0.985	-0.40	-6.93 – 6.14	0.905	-0.90	-7.42 – 5.61	0.785	-4.38	-10.47 – 1.71	0.158
Furosemide dose/40	3.78	2.64 - 4.92	**<0.001**	3.95	2.82 – 5.07	**<0.001**	3.80	2.65 – 4.94	**<0.001**	3.76	2.61 – 4.91	**<0.001**	2.29	1.09 – 3.49	**<0.001**
Anticoagulants															
No	Ref			Ref			Ref			Ref			Ref		
Dicumarinic	0.55	-2.76 - 3.87	0.743	0.87	-2.39 – 4.13	0.601	1.95	-1.31 – 5.21	0.240	2.27	-1.01 – 5.55	0.175	1.39	-1.62 – 4.41	0.364

DOAC	-3.50	-7.38 - 0.37	0.076	-2.58	-6.40 – 1.25	0.186	-1.54	-5.38 – 2.30	0.431	-1.66	-5.51 – 2.18	0.397	-1.35	-4.95 – 2.26	0.464
Cardiac rehabilitation															
No	Ref			Ref			Ref			Ref			Ref		
Yes	-5.82	-9.70 – -1.94	**0.003**	-6.17	-10.02 – -2.32	**0.002**	-5.92	-9.80 – -2.05	**0.003**	-5.38	-9.24 – -1.52	**0.006**	-2.90	-6.48 – 0.67	0.112
Nocturnal non-invasive ventilation															
No	Ref			Ref			Ref			Ref			Ref		
Yes	0.68	-2.71 - 4.07	0.694	1.10	-2.23 – 4.44	0.517	0.80	-2.52 – 4.12	0.635	0.96	-2.36 – 4.28	0.570	1.16	-1.96 – 4.28	0.465
Cardiac device															
None	Ref			Ref			Ref			Ref			Ref		
ICD	-6.69	-14.36 – 0.98	0.087	-4.37	-12.17 – 3.42	0.271	-2.83	-10.59 – 4.93	0.474	-3.84	-11.61 – 3.93	0.332	-5.29	-12.34 – 1.77	0.142
CRT-P	-10.39	-19.71 – -1.08	**0.029**	-9.49	-18.63 – -0.35	**0.042**	-8.56	-17.61 – 0.50	0.064	-9.44	-18.41 – -0.47	**0.039**	-13.55	-22.45 – -4.65	**0.003**
CRT-D	0.03	-8.39 – 8.45	0.995	1.34	-6.76 – 9.44	0.745	-0.09	-8.20 – 8.02	0.982	-0.28	-8.32 – 7.76	0.945	0.61	-6.75 – 7.97	0.872
Pacemaker	-0.20	-4.65 – 4.26	0.931	0.30	-4.07 – 4.67	0.892	-0.19	-4.57 – 4.18	0.931	-0.65	-5.03 – 3.74	0.772	-1.88	-5.87 – 2.11	0.354

ACEi, angiotensin-converting enzyme inhibitor; ARB, angiotensin-receptor blocker, ARNi, angiotensin receptor-neprilysin inhibitor; BB, beta-blocker; CI, 95% confidence interval; CRT-D, cardiac resynchronization therapy-defibrillator; CRT-P, cardiac resynchronization therapy-pacemaker; DOAC, direct-acting oral anticoagulant; ICD, implantable cardioverter-defibrillator; MLHFQ, Minnesota Living with Heart Failure Questionnaire; MRA, mineralocorticoid receptor antagonist; SGLT2i, sodium-glucose co-transporter 2 inhibitor. [a] All models were adjusted to time and time^2. [b] All variables were adjusted to model 1, age, sex, and low income. [c] All variables were adjusted to model 2, CKD, anaemia, CPD, anxiety, and depression. [d] All variables were adjusted to model 3 and selfcare adherence. [e] All variables were adjusted to model 4, NYHA, and NT-proBNP.
